# Tumor suppressor protein SMAR1 modulates the roughness of cell surface: combined AFM and SEM study

**DOI:** 10.1186/1471-2407-9-350

**Published:** 2009-10-02

**Authors:** Ruchika Kaul-Ghanekar, Sandeep Singh, Hitesh Mamgain, Archana Jalota-Badhwar, Kishore M Paknikar, Samit Chattopadhyay

**Affiliations:** 1 Agharkar Research Institute, Pune, Maharashtra, India; 2 National Center for Cell Science, Pune, Maharashtra, India; 3 Interactive Research School for Health Affairs (IRSHA), Bharati Vidyapeeth University, Pune, Maharashtra, India

## Abstract

**Background:**

Imaging tools such as scanning electron microscope (SEM) and atomic force microscope (AFM) can be used to produce high-resolution topographic images of biomedical specimens and hence are well suited for imaging alterations in cell morphology. We have studied the correlation of SMAR1 expression with cell surface smoothness in cell lines as well as in different grades of human breast cancer and mouse tumor sections.

**Methods:**

We validated knockdown and overexpression of SMAR1 using RT-PCR as well as Western blotting in human embryonic kidney (HEK) 293, human breast cancer (MCF-7) and mouse melanoma (B16F1) cell lines. The samples were then processed for cell surface roughness studies using atomic force microscopy (AFM) and scanning electron microscopy (SEM). The same samples were used for microarray analysis as well. Tumors sections from control and SMAR1 treated mice as well as tissues sections from different grades of human breast cancer on poly L-lysine coated slides were used for AFM and SEM studies.

**Results:**

Tumor sections from mice injected with melanoma cells showed pronounced surface roughness. In contrast, tumor sections obtained from nude mice that were first injected with melanoma cells followed by repeated injections of SMAR1-P44 peptide, exhibited relatively smoother surface profile. Interestingly, human breast cancer tissue sections that showed reduced SMAR1 expression exhibited increased surface roughness compared to the adjacent normal breast tissue. Our AFM data establishes that treatment of cells with SMAR1-P44 results into increase in cytoskeletal volume that is supported by comparative gene expression data showing an increase in the expression of specific cytoskeletal proteins compared to the control cells. Altogether, these findings indicate that tumor suppressor function of SMAR1 might be exhibited through smoothening of cell surface by regulating expression of cell surface proteins.

**Conclusion:**

Tumor suppressor protein SMAR1 might be used as a phenotypic differentiation marker between cancerous and non-cancerous cells.

## Background

Even though the current diagnosis of cancer depends mainly upon tissue biopsy inspection by routine optical microscopy [1], imaging tools such as scanning electron microscope (SEM) and atomic force microscope (AFM) can provide a better understanding of the surface details at the nanometer level. This will avoid possible ambiguity that may be imposed due to diffraction limitations (~250-300 nm) posed by light microscopy [2-5]. Despite high-resolution imaging obtained by electron microscopy, it lacks certain advantages, such as making precise structural measurements along the z-axis, that are provided by scanning probe microscopy, especially AFM. The two commonly used analytical techniques for high resolution surface imaging of materials is the SEM and AFM. Both these tools provide topographical information at a resolution far superior to optical methods [6,7]. Scanning electron microscope can be used to image topography of the sample or to determine the local composition, crystal structure, orientation, electrical and optical properties of the sample [8,9].

AFM is a probing-based instrument [6] that has gained significant importance in the recent years for studying biological samples at sub-nanometer scale in their natural aqueous environment [10-12]. The vertical resolution is mostly determined by the AFM scanner sensitivity, and is as high as 0.01 nm. Besides topography-based studies, due to its high sensitivity, AFM is being widely used to study receptor-ligand interactions, protein unfolding and cell adhesion [13-19]. AFM imaging is now being combined with fluorescence microscopy to study different cellular structures [20-22]. Due to application of low forces with minimal disruption to cells [23,24], AFM has been used to probe a number of inherent properties of microbial cells [25,26], mammalian cells and biomolecules apart from analyzing cellular mechanical strain and elasticity [15,27-33]. Apart from its use in probing cellular mechanics under physiological conditions, it has been recently used for nanomechanical analysis of live metastatic cancer cells from body fluids of patients suspected of suffering from various cancers [34].

In the present work, we are for the first time reporting the morphological differences between cancerous cell lines and cells overexpressing a tumor suppressor protein, SMAR1 (Scaffold/Matrix Associated Region binding protein 1) by utilising the nanoscale capabilities of both SEM and AFM. SMAR1, a matrix associated region binding protein (MARBP) [35] functions as a potent tumor suppressor through interaction with and activation of p53 ultimately resulting into G2/M arrest of the cells [36,37]. Interestingly, we have recently shown a drastic downregulation of SMAR1 in higher grades of human breast cancers [38]. Besides tumor suppressor function, SMAR1 controls T cell development through regulation of TCRβ transcription by modulating Eβ enhancer and TCRβ gene rearrangement [39,40]. SMAR1 interacts with a MARBP, Cux/CDP, and both synergistically regulate the TCRβ gene transcription [40]. Being a transcriptional repressor, SMAR1 has also been shown to repress cyclin D1 gene expression [41], whose higher expression is a hallmark in breast cancer. Since SMAR1 expression is decreased in majority of cancerous cells [36] and it has been shown to disrupt the tumor vasculature [42], we were interested in studying detectable phenotypic differences between the cells over expressing or under expressing SMAR1.

SEM and AFM imaging studies on HEK 293 as well as B16F1 cell lines, revealed a rough cell surface architecture. Interestingly, we found that upon overexpression of SMAR1, the cells showed smooth topographic features. These results were in concurrence with the data obtained from different grades of human breast cancer tissue sections. We have recently shown that with the advanced stages in breast cancer, SMAR1 expression level goes down [38]. Here, we have further illustrated that in advanced stages of breast cancer, the morphology of the cells become rough compared to the smooth surface feature of cells from tissue sections of adjacent normal globular breast area. Moreover, we also observed that topography of cells from tumor sections of mice injected with melanoma cells (B16F1) were rough compared to the cells from tumor sections of mice injected first with B16F1 cells followed by SMAR1-P44 peptide treatment. Further, by using AFM, we demonstrated that the cytoskeletal volume of cells (calculated from 'flooding/find hills' options in the WSxM software by Nanotec Electronica) overexpresssing SMAR1 was almost 3.5 folds more compared to the control cells. This is supported by data from microarray as well as mRNA expression studies that too exhibited increased expression of certain cytoskeletal proteins as well as few adhesion molecules in SMAR1 overexpressing cells. These results suggest that SMAR1 plays a key role in maintaining cellular topography through regulation of specific cytoskeletal proteins and this feature could be explored for phenotypic distinction between cancerous and non-cancerous cells.

## Methods

### Cell culture and transfection

Human embryonic kidney (HEK) 293, mouse melanoma (B16F1) and human breast cancer (MCF7) cell lines were grown in DMEM supplemented with 10% FBS in the presence of 5% CO_2 _at 37°C. Around 3 × 10^5 ^cells were plated on coverslips in 35 mm culture dishes. After 24 h, cells were transiently transfected using lipofectamine-2000 with SMAR1 cDNA full-length constructs from pBK-CMV (pBK-CMV-SMAR1) expression plasmid (1.0 μg) [36] or with 100 nM of custom synthesized SMAR1-siRNA oligonucleotides (Ambion Inc., Austin, TX) [37]. Forty eight hours post-transfection, the cells were processed for either SEM or AFM analysis.

### RT-PCR

The control cells as well as cells transfected with either full-length SMAR1 construct or SMAR1-siRNA oligonucleotide were harvested and total RNA was isolated that was subjected to cDNA synthesis following the manufacturer's instruction (Invitrogen). PCR was done using specific primers for SMAR1: F-5'-GCATTGAGGCCAAGCTGAAAGCTC-3' and R-5'-CGGAGTTCAGGGTGATGAGTGTGAC-3'; NEU: F-5'-ACCCATTAGCCAGACCACAG and R-5'-GGCTACTATCCCAACGACCA; MARK1: F-5'-CACTCTTCAGTCCCCTGCTC and R-5'-CAACTGTTGTGCTGCCAAGT; MYH10: F-5'-GTACCTTGCCCATGTTGCTT and R-5'-TTTTGCTTGAGCAACAGCAC; and β-actin F-5'-TACCACTGGCATCGTGATGGACT-3' and R-5'-TTTCTGCATCCTGTCGGAAAT-3'.

### Western Blotting

For western blotting, the control cells as well as cells transfected with either full-length SMAR1 construct or SMAR1-siRNA oligonucleotide were harvested, washed with 1× PBS and lysed in buffer as described earlier [37]. SMAR1 polyclonal antibody that was raised in house [41] was used for probing the blot.

### Histological sections

Mouse tumor sections used for SEM as well as AFM studies were obtained by establishing tumors in nude mice treated with either control B16F1 mouse melanoma cells or with SMAR1-P44 peptide [42]. Briefly, nude mice were subcutaneously injected with 2 × 10^6 ^B16F1 cells and once the tumors were clearly visible, SMAR1-P44 peptide was subcutaneously injected proximal to the tumor sites at a dose of 200 μg/ml/mouse three times a week. The treatment was continued for 4 weeks. For control experiments, B16F1 cells alone were subcutaneously injected into the mice and were monitored for tumor growth [42]. In each set of experiments, five mice were used and the mice were maintained under pathogen-free conditions. All the mice experiments were carried out with an approval from the Institutional (NCCS, Pune) Ethical as well as Biosafety Committee. Human breast cancer samples were classified into Infiltrating Ductal Carcinoma Grade I, II and III (IDC G I, II and III) by standard HE staining [38]. For comparison, adjacent normal globular tissue area was taken as normal control. Human breast cancer tissue sections were obtained from KEM hospital, Pune upon approval from Hospital Ethical Committee.

### Atomic force microscopy

For AFM measurements, the control cells as well as cells transfected with either SMAR1 or SMAR1-siRNA were fixed with 2.5% paraformaldehyde for 15 min, followed by washing twice with 1× PBS and twice with glass distilled water before AFM measurements (the last wash with glass distilled water was done because PBS forms crystal-like structures upon drying that results into artifacts). Breast carcinoma tissues used in the study had been obtained from KEM Hospital, Pune and the histological grading of tumor tissues was done following modified Bloom and Richardson guidelines. For AFM studies, the tumor sections in paraffin-embedded blocks were transferred to poly-L-lysine-coated glass slides and air-dried overnight at 37°C. They were dewaxed in xylene (three changes) and rehydrated in a graded series of decreasing ethanol concentration before subjecting for AFM imaging. The surface topography of cells as well as tumor histological sections obtained from nude mice and breast cancer patients was imaged using Multiview 1000™ AFM (NANONICS, Jerusalem, Israel) operating in amplitude modulated tapping mode. Glass fiber probes (NANONICS, Jerusalem, Israel), with a tip diameter (*Φ*) of 20 nm and nominal spring constant (*k*) of 10 N/m were used at a low resonance frequency (*ω*_*o*_) of 80 KHz. All AFM experiments were performed in air at ambient temperature. The surface of the cells was scanned in the x, y and z directions by a sharp tip, and moved by a piezoelectric translator. A laser beam reflected off the cantilever towards a four-segment photodiode sensed the deflection of the cantilever when the tip scanned the sample surface. All the images were acquired with a 512 × 512 data point resolution and a scan delay of 5 ms.

### Roughness analysis

The roughness of the surface of all the cells was analyzed as described earlier [43] by measuring the root mean square roughness, *R*_*rms*_, on the height image, which is defined as the standard deviation from the mean data plane of the h (height) values of the AFM images within a selected region on the cell surface:

where *h*_*i *_is the current height value; , the height of the mean data plane; and N, the number of points within the selected region of a given area. The roughness analysis was carried out on raw AFM images after normalization. For roughness analysis, four different cells from each group were taken and four different areas from each cell were chosen to calculate R_rms_. The areas chosen were 50 × 50 data points in dimension. The overall weighted mean surface roughness  was calculated as:

where  is the mean of the four values on each cell i, and each mean  value is weighted by a factor 1/σ_i_^2 ^where σ_i_^2 ^is its standard deviation. The overall standard deviation, σ, of the best estimate , is given by:

### Scanning electron microscopy

For SEM analysis, the control cells as well as those transfected with SMAR1 were processed for fixation wherein they were rinsed twice with phosphate buffered saline (PBS) followed by washing in buffer A (20 mM HEPES, 100 mM KCl, 2 mM MgCl_2_, pH 7.0). This was followed by incubation for 20 min with 2.5% p-formaldehyde in buffer A at 4°C. After extensive rinsing with buffer A, cells were dehydrated through increasing concentrations of ethanol (50, 70, 90 and 100%). Samples were dried and sputter coated with ~5 nm platinum. The field emission scanning electron microscope (JEOL, USA, JSM 6360) was operated at 10 kV.

### Cytoskeletal preparation for the imaging

HEK 293 cells (control and SMAR1 transfected), grown on coverslips were washed twice with Hank's Balanced Salt Solution (HBSS) solution. The cells were then treated for 5 min at room temperature with a buffer (10 mM Tris-HCl, pH 7.6, 0.14 M NaCl, 5 mM MgCl_2_, 4% polyethylene glycol 6000) containing 0.5% Triton X-100 detergent (Sigma) [44]. After the treatment, the cells were washed twice for 2 min in the buffer alone and then fixed in the buffer containing 1% formalin for 10 min. The treating solution was removed and the cells were washed twice with HBSS solution. For scanning in air, the cells were washed with MilliQ ultrapure water and dried under ambient conditions (24°C, 48% humidity). The cells were imaged immediately after treatment.

### Microarray analysis

Microarray was performed on B16F1 control and SMAR1-stably transfected cells as well as on HEK 293 control cells and those treated with SMAR1-P44 peptide [41]. The experiment was commercially performed by Agilent Genotypic Technology (Bangalore, India).

## Results

### Cells overexpressing SMAR1 exhibit smooth topography: SEM study

**Figure 1:**
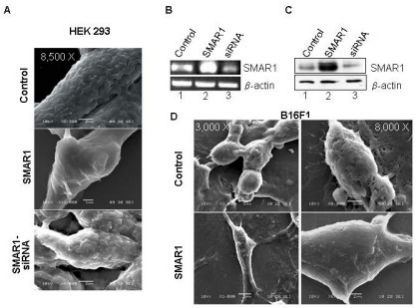
**Cells overexpressing SMAR1 exhibit smooth surface morphology as shown by SEM images**. (A) Images of control, SMAR1 and SMAR1-siRNA transfected (transient) human embryonic kidney cell line (HEK 293) (photographs taken at lower magnification of 3000 × and higher magnifications of 8,500 × for control and SMAR1-siRNA treated cells and 12000 × for SMAR1 treated cells); (B) RT-PCR and (C) Western blotting results showing the expression of SMAR1 in control, SMAR1 and SMAR1-siRNA transfected HEK 293 cell line wherein actin has been used as the loading control. (D) SEM images of control and SMAR1 stably transfected mouse melanoma cell line (B16F1) (photographs taken at lower magnifications of 1000 × and 3000 ×; and higher magnifications of 8000 × for control and 8,500 × for SMAR1 treated cells).

SMAR1 functions as a tumor suppressor protein that upregulates p53 along with a number of cell cycle regulatory proteins ultimately resulting into tumor retardation [36,37]. To determine if there are any discernible phenotypic differences between cancerous cells and cells overexpressing SMAR1, we utilized both SEM and AFM. Different cell lines: HEK 293, B16F1 and MCF7 (data not shown) were used in the study. The cells were transiently (HEK 293 and MCF7) or stably (B16F1) transfected with SMAR1 (full-length) and SMAR1-siRNA (in HEK 293 cells). The overexpression of SMAR1 was confirmed by RT-PCR (Fig. 1B) and western blot (Fig. 1C) in HEK 293 cells. SMAR1-siRNA treated cells showed a significantly reduced expression of SMAR1 compared to the control cells. SEM analysis of cells overexpressing SMAR1 (Fig. 1A, photographs taken at 8500 × magnification) exhibited smoother topography compared to either control cells or those treated with SMAR1-siRNA that exhibited rough surface features. Similar results were found in B16F1 cells wherein the morphology of control cells (Fig. 1D, upper panel; photographs taken at 3000 × and 8000 × magnification) was rough compared to SMAR1 stably transfected cells (Fig. 1D, lower panel; photographs taken at 3000 × and 8500 × magnification). MCF7 cells also exhibited similar results wherein the morphology of SMAR1 treated cells was smoother compared to the control cells (data not shown).

**Figure 2:**
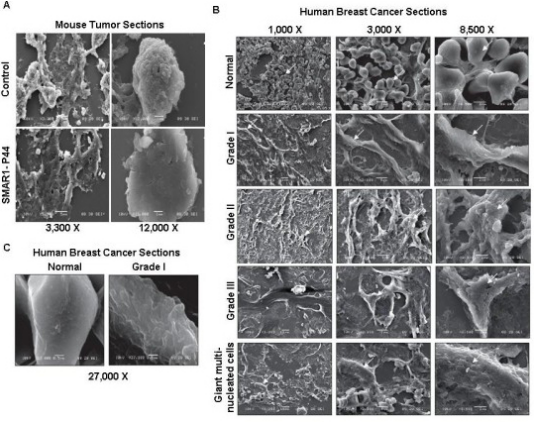
**Mice and human tumor tissue sections exhibit rough surface topography compared to the normal control tissue sections**. SEM images of (A) tumor sections from nude mice treated with control B16F1 cells (photographs taken at lower magnifications of 3,300 × and 4000 × and higher magnification of 12,000 ×) and SMAR1-P44 (photographs taken at lower magnification of 3,300 × and 7,500 × and higher magnification of 12,000 ×), (B) sections from human breast cancer cells of different patients. Images from normal breast tissue section and various grades of breast cancer cells: Grade I, Grade II, Grade III and giant multinucleate cells are shown. Photographs have been taken at 1000 ×, 3000 × and 8500 × magnification for all the tissue sections except for the normal breast tissue as well as Grade I tumor section wherein the highest magnification used was 27,000 ×.

Since SMAR1 is downregulated in majority of cancers [36], we determined the morphology of cells isolated from tumors raised in nude mice and compared them with tumors isolated from SMAR1-chimera peptide treated mice. For this, nude mice were injected with mouse melanoma cells (B16F1) to develop subcutaneous tumors [42]. In the test group, the mice that had been injected with B16F1 cells were given repeated doses of SMAR1-P44 peptide to retard the tumor growth. SMAR1-P44 is a 33-mer peptide of SMAR1 that retains the tumor suppressor function of full-length protein [42]. Since the treated mice exhibited inhibition of tumor growth, we used tissue sections from those tumors for SEM as well as AFM studies. As expected, SEM study showed that tumor sections from SMAR1-P44 treated mice (Fig. 2A, lower panel, photographs taken at 3300 × and 12000 × magnification) exhibited much smoother topography compared to the tumor sections from control mice (Fig. 2A, upper panel, photographs taken at 3300 × and 12000 × magnification). Since SMAR1 expression is known to be downmodulated in different cell lines [36], particularly breast cancer cells [38], we were interested in detecting phenotypic differences between normal and cancerous cells isolated from human subjects. For this, we obtained tissue sections from normal adjacent globular breast tissue and cancerous tissue from patients having different grades of breast cancer (Infiltrating Ductal Carcinoma (IDC) Grade I, II and III) [38]. It was observed that with increasing stages of cancer (from Grade I to Grade III), the surface roughness of the cells increased compared to the sections from normal breast tissue that had much smoother topography (Fig. 2B). Moreover, it was observed that the giant multinucleate cells exhibited significantly rough morphology compared to either normal or different grade tumor sections. Photographs have been taken at 1000 ×, 3000 × and 8500 × magnification for all the tissue sections. For better clarification of the difference between cell surface roughness of normal breast tissue versus grade I breast tumor, we have taken pictures at 27000 × magnification showing a remarkable difference in the cell surface topology between the two (Fig. 2C).

### AFM study confirms the smooth surface profile of SMAR1 overexpressing cells

AFM provides tri-dimensional high-resolution information about the surface of a sample, and thus cannot give any direct information about the interior of a cell. The AFM shows the topography (height), processed, 3D and profile images of the cells. The height range over the whole cells is usually several microns, so the 3D topography image shows the overall height of the cells. The processed signal image gives the information about the fine details of the cell structure. The processed images clearly show the smallest and sharpest features of the cell surface.

**Figure 3:**
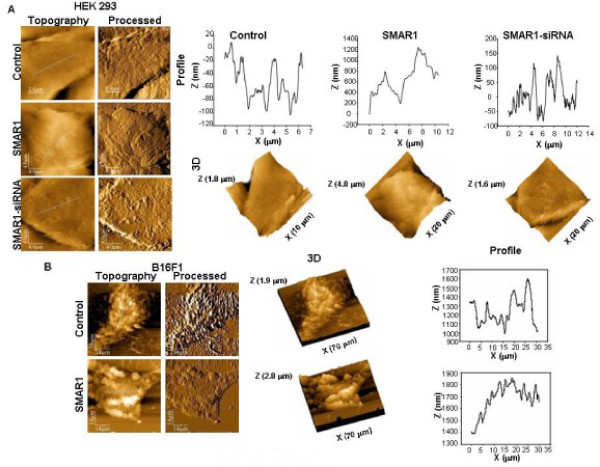
**SMAR1 overexpressing cells exhibit smooth surface profile**. AFM images of (A) human embryonic kidney (HEK 293) cell line. Height (topography), processed, 3D and profile (of line shown in topography image) of control (x-y range 10 × 10 μm, z range 1.8 μm); SMAR1 (x-y range 20 × 20 μm, z range 4.8 μm) and SMAR1-siRNA (x-y range 20 × 20 μm, z range 1.6 μm) are shown. (B) AFM images of mouse melanoma (B16F1) cell line. Height, processed, 3D and profile (of line shown in height image) of control (x-y range 70 × 70 μm, z range 1.9 μm) and SMAR1 (x-y range 70 × 70 μm, z range 2.8 μm) are shown.

**Figure 4:**
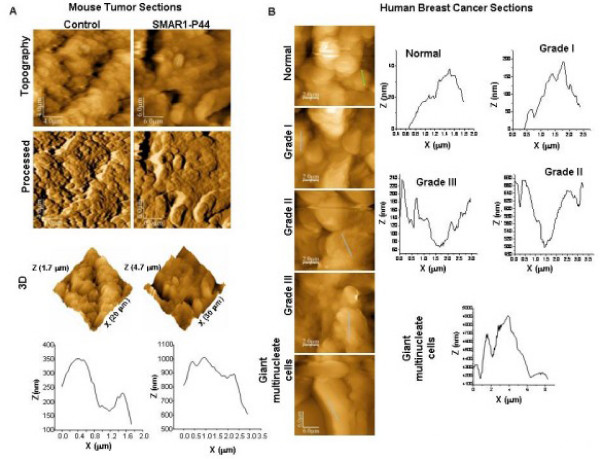
**AFM images showing smooth topography in normal tissues compared to the cancerous tissue sections**. (A) AFM images of tumor sections from nude mice. Height, processed, 3D and profile (of line shown in height image) of tumor sections from control (x-y range 20 × 20 μm, z range 1.7 μm) and SMAR1-P44 (x-y range 30 × 30 μm, z range 4.7 μm) treated mice are shown. (B) AFM images of human breast cancer sections. Height image and profile (line shown in height image) of tumor sections from normal breast tissue (x-y range 10 × 10 μm, z range 0.84 μm); Grade I (x-y range 10 × 10 μm, z range 1.17 μm), Grade II (x-y range 10 × 10 μm, z range 1.4 μm) and Grade III (x-y range 10 × 10 μm, z range 2.65 μm) tumors and Giant multinucleated cells (x-y range 30 × 30 μm, z range 10.8 μm) are shown.

It was observed that in each cell line tested, HEK 293 (Fig. 3A) and B16F1 (Fig. 3B), the surface topography of control and SMAR1-siRNA treated (in HEK 293 cells only) was rougher when compared to SMAR1-treated cells (Fig. 3A, 3B). The profile images of control and siRNA treated cells were irregular whereas SMAR1-treated cells exhibited relatively regular surface profile with small overall height modulations. Similarly, tumor sections from mice injected with B16F1 cells showed pronounced surface roughness compared to the tissue sections derived from mice treated with SMAR1-P44 peptide (Fig. 4A).

Earlier it been has shown that SMAR1 is drastically down-regulated in higher grades of breast cancer [38]. The same sections corresponding to various grades of breast cancer were used to study the difference in surface topology of different grades of breast cancer. Upon comparison of tissue sections from normal breast tissue with those from different grades of breast cancer (Fig. 4B), we observed that the former exhibited smoother surface architecture compared to the latter.

The statistical distribution of the mean roughness  (i = 1, 2, 3, 4) was determined on control as well as SMAR1-treated cells from each cell line [HEK 293, B16F1 and MCF7 (data not shown)] (Fig. 3A, 3B, respectively), as well as histological sections (mouse as well as human; Fig. 4A and 4B, respectively), which showed that the mean roughness was centered on the weighted mean surface roughness, , as reported in Tables 1 and 2. Control cells of HEK 293 and B16F1 showed 1.7-fold and 1.4-fold, increase in surface roughness, respectively, compared to their respective SMAR1-overexpressed cells (Table 1). Likewise, tumor sections from control mice exhibited 2-fold increase in surface roughness than SMAR1-P44 peptide treated mice (Table 1). Interestingly, in histological sections from human breast cancer tissue, it was observed that with increase in the grade of cancer, there was an increase in the surface roughness (Table 2) wherein Grade III stage cells showed 4.4-fold increase in surface roughness compared to the control cells. Moreover, the tissue sections from giant multinucleate cells depicted a marked increase (24.7-fold) in surface roughness compared to the control cells as observed in Table 2.

**Table 1:** Weighted mean surface roughness data of control, SMAR1 overexpressing and SMAR1 siRNA treated cells.

Samples	Control Roughness (nm)*,	SMAR1 Roughness (nm)*,	SMAR1-siRNA Roughness (nm)*,
HEK 293 cell line	49.0154 ± 0.0324	29.6985 ± 0.0533	50.2835 ± 0.0321
B16F1 cell line	132.871 ± 0.00368	97.349 ± 0.0756	-
Mouse Tumor Sections	124.3178 ± 8.3362e-3	62.1869 ± 0.0103 (SMAR1-P44 treated)	-

*The surface areas chosen to calculate the cell surface roughness were 50 × 50 data points for all the cells and tissue sections.  represents the weighted mean surface roughness. The values for the surface roughness of the cells were calculated over 4 different cells and are given as the weighted mean ± standard deviation

**Table 2:** Weighted mean surface roughness data of human breast tissue sections from normal cells as well as different grades of cancer.

Human Breast Tissue Sections	Roughness (nm)*,
Normal	24.6404 ± 0.1254
Grade I	33.8073 ± 0.0467
Grade II	61.4068 ± 0.0118
Grade III	109.0707 ± 2.9733e-3
Giant Multinucleate cells	606.7377 ± 2.8342e-3

*The surface areas chosen to calculate the cell surface roughness were 50 × 50 data points for all the tissue sections.  represents the weighted mean surface roughness. The values for the surface roughness of the cells were calculated over 4 different cells and are given as the weighted mean ± standard deviation.

### Increase in the cytoskeleton volume of SMAR1 overexpressing cells

Cancer is a disease that results due to malfunctioning of a normal cell leading to abnormal proliferation of cells that ultimately disrupt the tissue organization. The shape and mechanical rigidity of a cell is determined by the internal biopolymeric protein scaffolding that constitutes the essential components of the cell cytoskeleton [31,45,46]. Altered protein structure of cancer cells also modifies the shape, rigidity as well as motility of the cells, thus affecting the cellular cytoskeleton.

**Figure 5:**
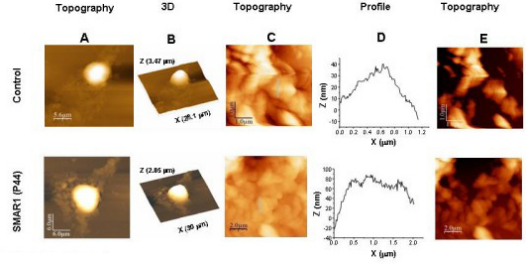
**SMAR1 overexpression results into increased cytoskeletal volume**. AFM images of cellular cytoskeleton of HEK 293 control cells as well as those treated with SMAR1-P44 peptide. Height images of a group of cells (A) and a single cell (B) are shown whose 3D (C) is also depicted. Height (D) and profile (E) images of a zoomed area within the single cell are shown in both control and SMAR1-P44 treated cells. Using flooding/find hills option of free software WSxM (to calculate the volume of the cellular cytoskeleton), the zoomed image showing the volume of cytoskeleton (F) is more in SMAR1-P44 treated cells than the control cells.

Our results show that SMAR1 maintains the cellular morphology that depends upon the underlying cytoskeletal framework of the cells. Thus, we were interested in detecting differences, between the cytoskeletal volume of control and SMAR1-overexpressing cells. The cytoskeletons of control and SMAR1 overexpressing cells were prepared for AFM study as mentioned in Materials and Methods [44]. Before quantifying the cytoskeletal volume, the topography (Fig. 5A) and 3D image (Fig. 5B) of entire cytoskeletal framework of control cells and cells treated with SMAR1-P44 peptide was analysed wherein the nucleus appeared distinct and raised. The topography and profile images of the cytoskeletal region (excluding the nuclear region) could be observed (Fig. 5C and 5D) that exhibited pronounced differences between the control and SMAR1-P44 peptide treated cells. The cytoskeletal volume was quantified by using the free software WSxM by Nanotec Electronica (version 4.0 develop 3.4). After drying the sample, the cytoskeleton framework collapses to the surface and it was assumed that the volume of the collapsed cytoskeleton is equivalent to the volume of cytoskeletal fibers and insoluble components of the endoplasmic reticulum [44]. The calculations of the volume were done over the area away from the nucleus by first removing any overall tilt of the image, and then by using "flooding/find hills" option of the software. For comparing the cytoskeleton of control HEK 293 cells with that of SMAR1-P44 treated cells, cytoskeletal volume was normalized by dividing the volume by the area it occupies (Table 3). Interestingly, we found that the volume of cellular cytoskeleton (topographic image shown in Fig. 5E), calculated as mentioned above, was almost 3.5 times more in SMAR1 overexpressing cells compared to the control cells.

**Table 3:** Cytoskeleton volume/area ratio calculated for control versus SMAR1 (P44) peptide treated cells.

HEK 293 cells	Flooded Area (A)	Flooded Volume (V)	Ratio [V/A]
Control	13.4062 μm^2^	3.9723e+010 Å^2^	29.63
SMAR1-P44 treated	76.4601 μm^2^	7.94947e+011 Å^2^	103.96

## Discussion

Alterations in the physical properties, particularly cell elasticity, of tissue cells have been recently considered as an indication of disease [47-49] and thus has evolved as a phenotypic marker for cellular events associated with cell adhesion and cytoskeletal organization [47,50-52]. Several studies have shown that with increase in metastatic efficiency in human cancer cell lines, there is a reduction in the cell stiffness (elasticity) [28-30]. It was also recently reported that the cell stiffness, measured by force-displacement curves, of metatstatic cancer cells taken from the body (pleural) fluids of patients was softer than the benign cells lining the body cavity [34].

In this study, we have for the first time studied the topography of cells overexpressing SMAR1, a tumor suppressor protein and compared with the control cells that express less SMAR1. This protein has been earlier shown to interact with and activate tumor suppressor protein p53, which in turn activates the downstream effector, p21. This results into activation of several cell cycle proteins, which ultimately result into retardation of cells at G2/M stage [36]. Since SMAR1 retards tumor growth, we were interested in investigating its role in the regulation of cell morphology. Even though it is possible to correlate the shape deformation with the mechanical or biochemical pathway that induced the change, an appropriate quantitative morphological analysis can help in understanding how the modifications take place. We used both AFM and SEM tools to investigate the topography of cells overexpressing SMAR1. Interestingly, from both the microscopic studies we found that SMAR1 induced the cells to exhibit smooth topography compared to rough surface profiles of either control or SMAR1-siRNA treated cells. Experiments were performed on different cell lines as well as on tumor sections from mice and human breast cancer sections. In all the cell lines, the control cells exhibited significant surface roughness compared to SMAR1 overexpressed cells. The tumor sections from mice injected with B16F1 melanoma cells also showed rougher surface feature compared to those treated with SMAR1-P44 peptide. This 44-mer chimeric peptide contains 11-mer TAT PTD -domain fused with 33-mer core region of SMAR1 that retains the tumor suppressor activity of the full-length protein [42]. Thus, compared to the cell lines that were transfected with the full-length SMAR1, even the SMAR1-P44 chimera peptide was sufficient to impart smooth topography to otherwise rough control cells. The difference in the surface profile was more significant in tumor sections obtained from SMAR1-P44-treated mice, which exhibited a 2-fold decrease in surface roughness compared to the control mice. Our results were further validated by using clinical samples of breast tumor sections obtained from human patients with different grades of cancer. The tissue sections demonstrated that in advanced stages of breast cancer (Grade III) there was a 4.4-fold increase whereas in giant multinucleate cells, there was an astounding 24.7-fold increase in the surface roughness compared to the control cells from normal breast tissue. These results correlate with the reported observation that with increase in the grade of cancer in these tissue sections, there is a significant reduction in the expression of SMAR1 protein [38]. Thus, with decrease in the expression of SMAR1, the surface roughness of cells increases, thereby implicating the role of SMAR1 in the regulation of cellular morphology.

Current detection of cancer relies on qualitative morphological analyses of change in shape of cells that result from biochemical alterations, such as cytoskeletal remodeling [53]. In cancerous cells, there is an overall dysregulation of many proteins including the cytoskeleton proteins thereby resulting into overall disturbance of cellular architecture as well as rearrangement of the dynamic structures involved in cell division and motility [30]. Thus, the dynamic cytoskeleton reorganization has become important with respect to alterations in cell morphology, motility, adhesion and invasion [54,55]. The cytoskeleton framework of cells is mainly composed of microfilaments, microtubules and intermediate filaments, which together with cell surface receptors and ECM proteins help in regulating cellular morphology. All these proteins together not only help in maintaining the cell shape but also in cell adhesion and elongation, thereby organizing the cellular surface into structural and functional microdomains [56-59]. Since the cellular morphology is governed by the underlying cytoskeleton framework, we studied the cytoskeleton of control as well as SMAR1-overexpressing cells. Presently, there are three main techniques for studying the cellular cytoskeleton: TEM, immunofluorescence microscopy, and recently developed, transmission X-ray microscopy [44]. Besides studying the morphology, atomic force microscopy is a newer technique that has been recently used to visualize cellular cytoskeleton as well [44]. Our AFM data clearly shows a marked increase in the volume of cellular cytoskeleton in SMAR1 overexpressing cells. Interestingly, these results are supported by cDNA microarray data wherein we have compared the data of HEK 293 control cells with those treated with SMAR1-P44 peptide [see Additional file 1, Supplementary Table S1] as well as control B16F1 cells versus cells stably expressing SMAR1 [GEO Accession Number: GSM444125; see Additional file 1, Supplementary Table S2]. We observed that compared to the control cells, genes particularly coding for the proteins involved in the regulation of cellular architecture were upregulated in cells overexpressing SMAR1. The microarray data was also supported by mRNA expression studies in HEK 293 cells [see Additional file 1, Supplementary Fig S1] that demonstrated upregulation of specific adhesion proteins such as neurexin (NEU) as well as cytoskeletal proteins such as MAP/microtubule affinity-regulating kinase 1 (MARK1) and Myosin heavy polypeptide 10 (MYH10) in cells overexpressing SMAR1 compared to either control cells or those transfected with SMAR1-siRNA. Further, using confocal microscopy we observed that SMAR1 overexpression/knockdown does not affect the expression or localization of actin and β-tubulin [see Additional file 1, Supplementary Fig. S2 A and B, respectively] proteins. On the other hand, SMAR1 overexpression leads to upregulation of fibronectin and vinculin proteins while its knockdown leads to downregulation of their expression [see Additional file 1, Supplementary Fig. S2 C and D, respectively]. Taken together, these results imply that the increased cytoskeletal volume in SMAR1 overexpressing cells as shown by AFM could be probably due to the increase in the expression of cytoskeletal proteins. Thus, SMAR1 seems to restore the defective expression of certain architectural proteins that may be responsible for imparting smooth topography to otherwise rough cancerous cells.

Defects in the structure of the cytoskeleton influence a number of diseases, including different types of tumors [30]. The response of cells to structural and molecular alterations induced by the onset and progression of diseases also alter their elastic and viscoelastic properties. Alterations in cytoskeleton framework of cells are accompanied by changes in cell shape and mobility [33,60]. Though our data clearly indicates that SMAR1 modifies the cellular cytoskeleton that may be responsible for rendering smooth topology to the cells but further molecular events that induce this potential change in surface roughness need to be explored in detail.

## Conclusion

The morphological changes in cell and tissue have remained the benchmark for cancer diagnosis. Variations in nucleus size, shape and cell morphology are possibly related to the functional alterations in cancer cells and may offer crucial evidence to a particular tumor type and successful treatment. The powerful imaging tools such as SEM and AFM are complementary and can provide valuable information about the surface details of cells, particularly cancerous cells. These techniques overlap in their capabilities to provide nanometer scale lateral information, however, deviating from the fact that the AFM can provide tri-dimensional mapping of the surface resulting into generation of true topographic data with vertical resolution down to the subnanometer range. The high-resolution methods employed in the present work for investigation of morphological alterations between cells over- and under-expressing tumor suppressor protein, SMAR1, provide a method for differentiation between non-cancerous and cancerous phenotype, respectively. Thus, in conclusion our studies suggest that smooth cell surface profile for non-cancerous cells could be attributed to increased expression of SMAR1 whereas rough cellular morphology of cancerous cells could be attributed towards decreased expression of SMAR1 in the latter.

## List of abbreviations used

SMAR1: Scaffold/Matrix Associated Region 1 binding protein; AFM: Atomic Force Microscope; SEM: Scanning Electron Microscope; HEK 293: human embryonic kidney cell line; MCF-7: human breast cancer cell line; B16F1: mouse melanoma cell line.

## Competing interests

The authors declare that they have no competing interests.

## Authors' contributions

RKG and SC participated in the concept and design of the study. RKG, SS, HM and AJB have done the experiments. RKG, HM and KMP have carried out the AFM and SEM studies. AJB and SS have done the cell culture experiments. AJB has done mice experiments. All authors read and approved the final version of the manuscript.

## Pre-publication history

The pre-publication history for this paper can be accessed here:

http://www.biomedcentral.com/1471-2407/9/350/prepub

## Supplementary Material

Additional file 1 **Expression profiling of cytoskeletal proteins**. Data providing mRNA expression and microarray profiling of various cytoskeletal proteins. It includes supplementary figure S1 and supplementary tables S1 and S2. Click here for file
