# Supplementary material for: Tumor suppressor protein SMAR1 modulates the roughness of cell surface: combined AFM and SEM study
Source: BMC Cancer. 2009 Oct 2;9:350. doi: 10.1186/1471-2407-9-350 (PMC2765988; doi:10.1186/1471-2407-9-350)

**Additional files**

**Tumor suppressor protein SMAR1 modulates the roughness of cell surface: combined AFM and SEM study**

**Supplementary figures**

**Supplementary Figure S1.**

**mRNA expression of cytoskeletal proteins.** (*A*) RT-PCR showing mRNA expression of some of the cytoskeletal proteins: Neurexin (NEU), MAP/microtubule affinity-regulating kinase 1 (MARK1) and Myosin heavy polypeptide 10 (MYH10) in control, SMAR1 and SMAR1-siRNA transfected cells. (B) Graphical representation of relative expression levels of cytoskeltal genes.

**Supplementary Figure S2.**

**Confocal analysis of cytoskeleton proteins.**  MCF7 cells were transfected with GFP-SMAR1 or SMAR1 *si*RNA and processed for confocal analysis of few cytoskeletal proteins. SMAR1 is stained with FITC (green color) while Actin, β-tubulin, Fibronectin and Vinculin were stained with Cy3 (red color). SMAR1 overexpression or knockdown does not effect expression or localization of Actin (A) or β-tubulin (B). On the other hand, SMAR1 overexpression leads to upregulation of Fibronectin (C) and Vinculin (D) while its knockdown causes downregulation of their expression. Blue color shown in the figure represents the nucleus stained with DAPI.

**Supplementary tables**

**Supplemental Table S1. Microarray Analysis of Human Embryonic Kidney Cell Line (HEK 293) Control versus P44 peptide treated cells**

| **Protein** | **Symbol** | **Unigene** | **Function** | **Ratio*** |
| --- | --- | --- | --- | --- |
| Fibronectin type III domain containing 5 | FNDC5 | Hs.524234 | actin cytoskeleton regulation | **0.48** |
| Myosin, heavy polypeptide 10 | MYH10 | Hs.16355 | actin binding | **0.49** |
| MAP/microtubule affinity-regulating kinase 1 | MARK1 | Hs.497806 | cytoskeleton organization and biogenesis | **0.49** |
| Platelet-activating factor acetylhydrolase, isoform Ib | PAFAH1B1 | Hs.77318 | cytoskeleton, dynein binding | **0.33** |
| Syndecan 1 | SDC1 | Hs.224607 | cytoskeletal protein binding | **0.50** |
| CDC42 effector protein 3 | CDC42EP3 | Hs.369574 | cytoskeletal regulatory protein binding | **0.43** |
| Lymphocyte cytosolic protein 1 (L-plastin) | LCP1 | Hs.381099 | actin binding | **0.36** |
| Neurexin 1 | NRXN1 | Hs.468505 | cell adhesion | **0.36** |
| Integrin, beta 8 | ITGB8 | Hs.285724 | cell-matrix adhesion | **0.50** |

Altered gene expression in human embryonic kidney cell line (293) was identified by cDNA microarray analysis

*Relevant expression of selected genes significant for cytoskeleton regulation as well as adhesion is reported as the ratio of control to SMAR1 peptide treated cells.

**Supplemental Table S2. Microarray Analysis of Mouse Melanoma cell line (b16f1) control versus smar1 stably transfected cells**

| **Protein** | **Symbol** | **Unigene** | **Function** | **Ratio*** |
| --- | --- | --- | --- | --- |
| Fibronectin 1 | Fn1 | Mm.193099 | Regulation of actin cytoskeleton | **0.43** |
| Myosin IXa | Dmxl2 | Mm.249545 | actin binding | **0.47** |
| Microtubule-associated protein 6 | Mtap6 | Mm.154087 | Regulation of actin cytoskeleton | **0.48** |
| tau tubulin kinase 1 | Ttbk1 | Mm.275698 | microtubule associated | **0.24** |
| Syndecan binding protein (syntenin) | Sdcbp2 | Mm.32068 | cytoskeletal protein binding | **0.29** |
| Restin | Rsn | Mm.241109 | intermediate filament, microtubule cytoskeleton | **0.49** |
| Integrin alpha 5 (fibronectin receptor alpha) | Itga5 | Mm.16234 | Focal adhesion, Regulation of actin cytoskeleton | **0.37** |
| Thioredoxin interacting protein | Txnip | Mm.275340 | Regulation of actin cytoskeleton | **0.37** |
| Fibroblast growth factor 1 | Fgf1 | Mm.241282 | Regulation of actin cytoskeleton | **0.38** |
| Vinculin | Vcl | Mm.279361 | Focal adhesion, Regulation of actin cytoskeleton | **0.44** |
| Cytoplasmic FMR1 interacting protein 2 | Cyfip2 | Mm.154358 | Regulation of actin cytoskeleton | **0.44** |
| fascin homolog 2 | Fscn2 | Mm.134230 | actin-bundling protein | **0.31** |
| laminin, alpha 5 | Lama5 | Mm.4339 | Cell Communication, Focal adhesion | **0.20** |
| procollagen IV, a-1 | Col4a1 | Mm.738 | Cell Communication, Focal adhesion | **0.41** |
| procollagen, type V, alpha1 | Col5a1 | Mm.7281 | Cell Communication, Focal adhesion | **0.22** |

Altered gene expression in mouse melanoma cell line was identified by cDNA microarray analysis

*Relevant expression of selected genes significant for cytoskeleton regulation as well as adhesion is reported as the ratio of control to SMAR1 treated cells

**Supplemental FigureS1**

**
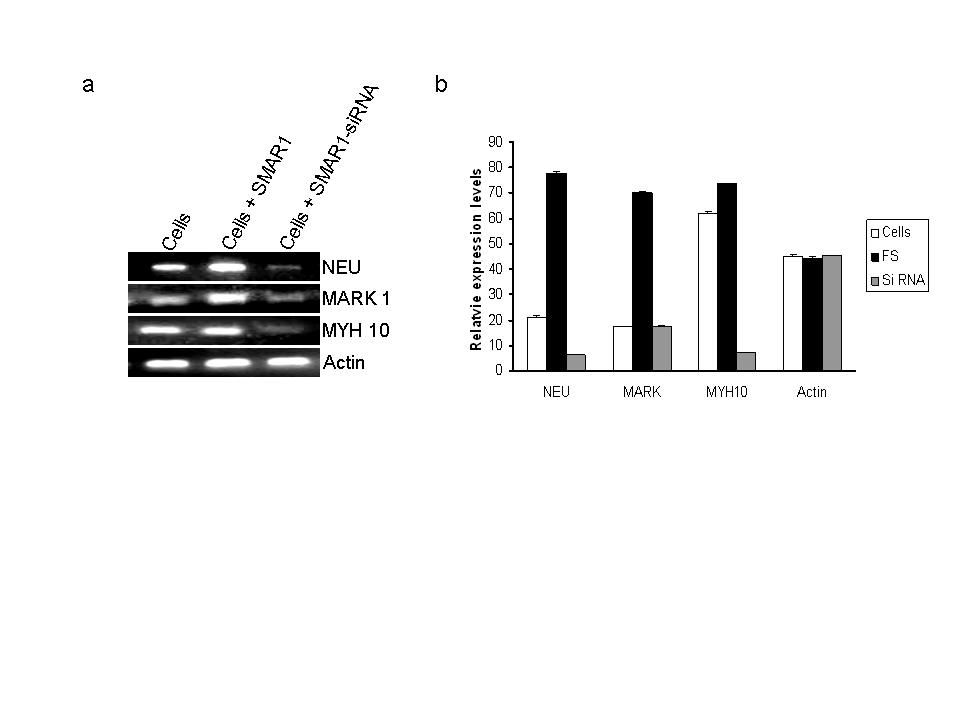
**

**Supplemental Figure S2**


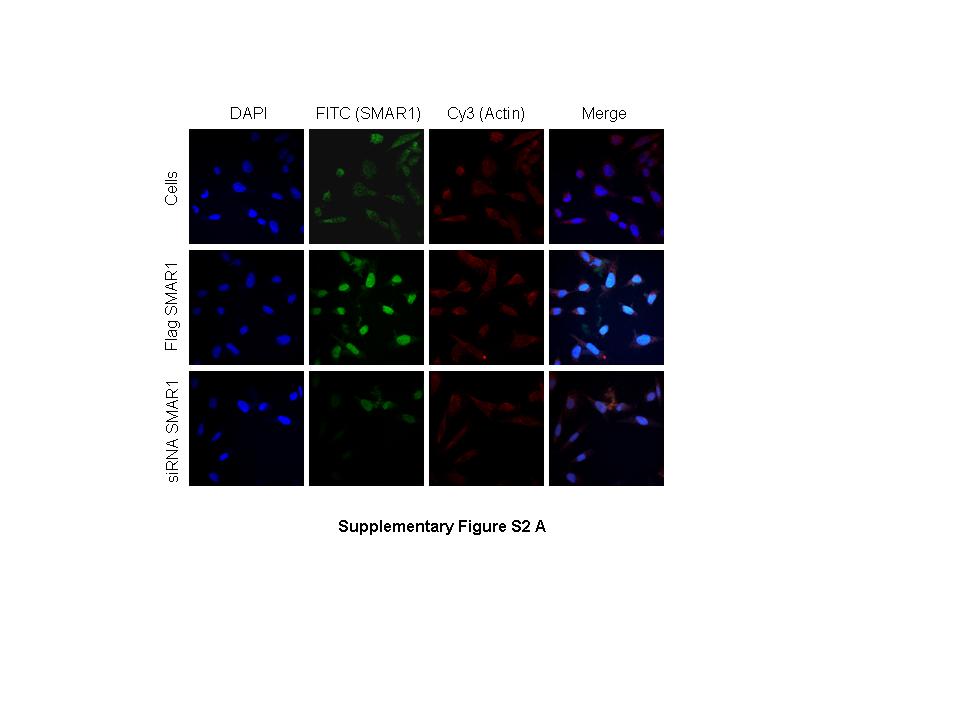

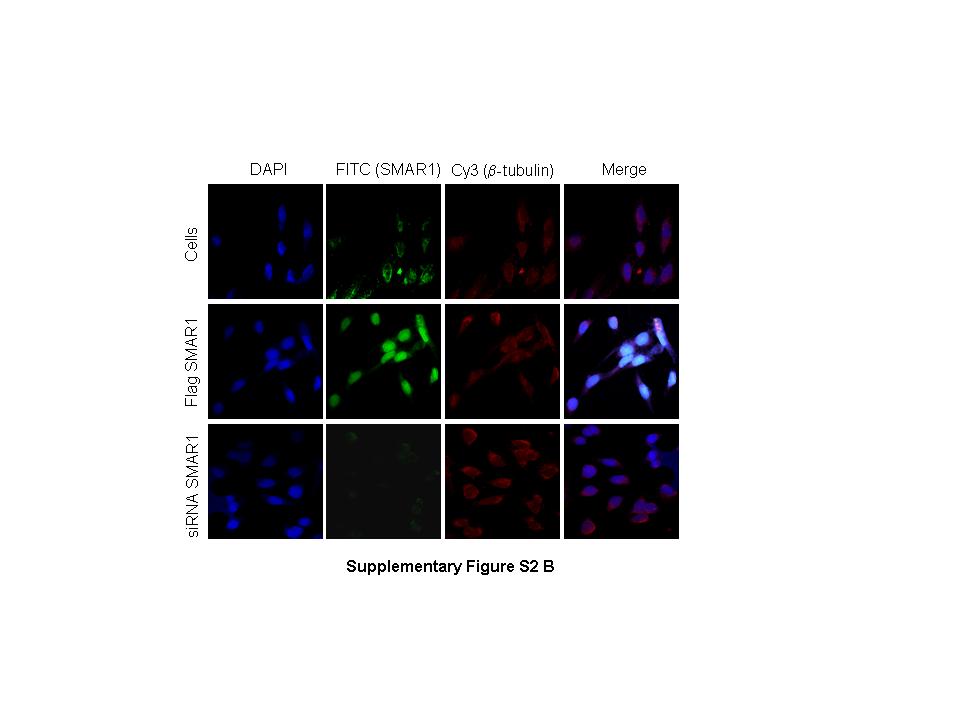

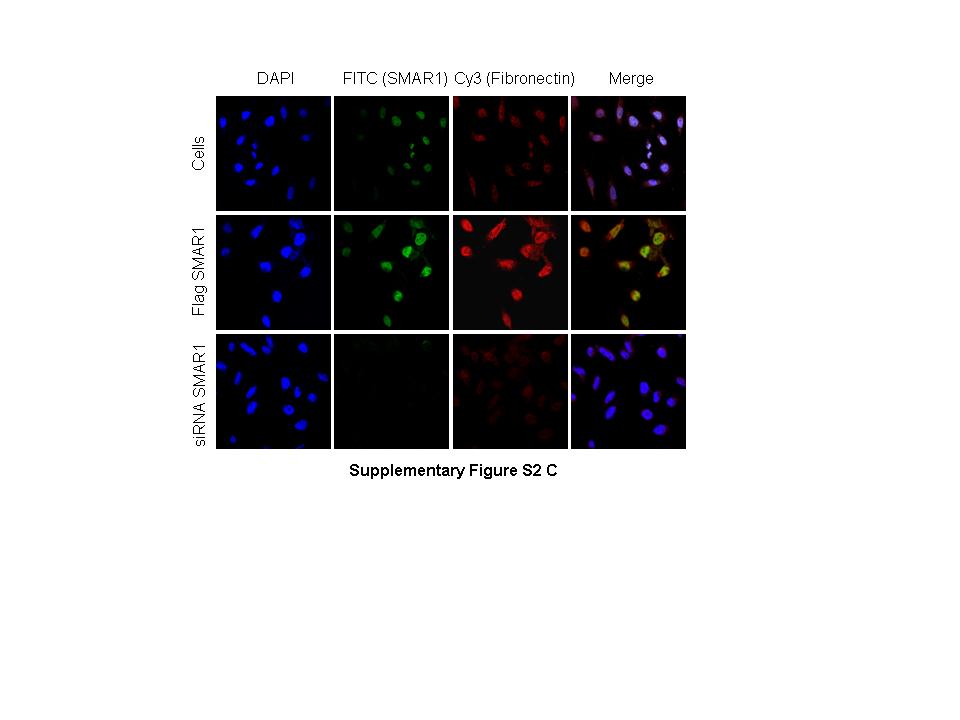

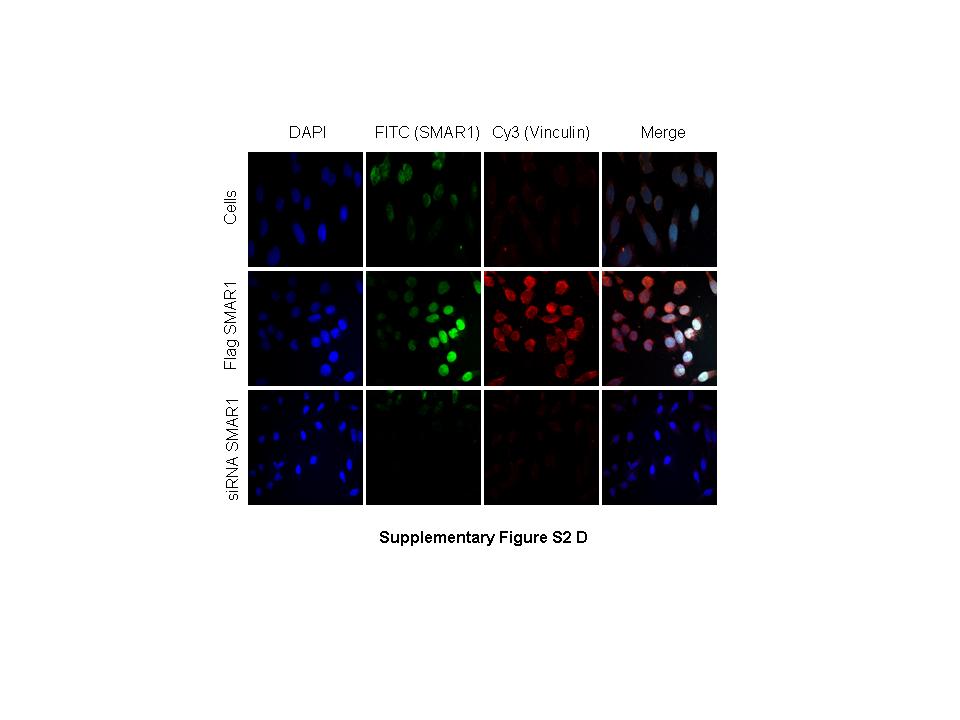

Supplement: Additional file 1 — Expression profiling of cytoskeletal proteins. Data providing mRNA expression and microarray profiling of various cytoskeletal proteins. It includes supplementary figure S1 and supplementary tables S1 and S2. [file 1471-2407-9-350-S1.DOC]
